# Measuring Structural Racism to Improve Older Adult Health Using Publicly Derived Data

**DOI:** 10.1093/geroni/igaf122.1474

**Published:** 2025-12-31

**Authors:** Karen Bandeen-Roche, Erik Westlund, Sierra Grey-Coker, Boeun Kim, Roland Thorpe, Sarah Szanton

**Affiliations:** Johns Hopkins Center on Aging and Health, Baltimore, Maryland, United States; Johns Hopkins Bloomberg School of Public Health, Baltimore, Maryland, United States; Johns Hopkins University, Baltimore, Maryland, United States; University of Iowa, Iowa City, Iowa, United States; Johns Hopkins Bloomberg School of Public Health, Baltimore, Maryland, United States; Johns Hopkins University, Baltimore, Maryland, United States

## Abstract

Racial disparities in older Americans’ health have been extensively documented, hence must urgently be investigated and addressed. Among potential causes, this presentation concerns structural racism— “the totality of ways in which societies foster [racial] discrimination, via mutually reinforcing [inequitable] systems (e.g., in housing, education, …, etc.),” (Bailey ZD et al., 2017). It aims to measure structural racism using publicly available data that were freely downloaded from websites of government agencies or social justice organizations, and that characterized geographical units nationwide. We report a partial hierarchy of models by which discrimination in mutually reinforcing systems might erode health. Five county-level indicators measuring Black-White disparity in healthcare, education, and policing were analyzed to illustrate the modeling framework and attendant data challenges. Factor analysis and cluster (latent profile) analysis agreed in identifying two major dimensions underlying the indicators: One in which disparities in preventive health care, educational progression, and remaining un-incarcerated tended to be lower or higher all together; and a second where benefits and harms coexisted, most strongly contrasting education and incarceration. Correlation of a z-score index of the indicators with the first factor exceeded 0.97 but was negligible with the second factor; clusters distinguished individuals whose indices were similar and vice versa. Correlations among measures differed substantially by geographic regions, indicating different systemic reinforcement networks. These findings suggest that meaningful signals can be derived from publicly derived data and a modeling hierarchy adds value over simple-sum indices. It aims to equip researchers with improved measures to elucidate, and address, health inequities.

